# 

**Published:** 1918-06-15

**Authors:** 


### CONTENTS.

| | Page |
|---|---|
| **Food Rationing and National Efficiency** ... | 217 |
| **Industrial Accidents in War-Time** ... ... | 218 |
| **Hospital and Institutional News** ... ... ... | 219 |
| A Testimonial to Dr. Rhodes—Hospital Secretary's Death in Mesopotamia—The New Superintendent of the Devonshire Hospital, Buxton—The Prince of Wales' Birthday—A Potted Report—Reduction of the Hospital Jam Ration—Order of the British Empire—A School of Army Sanitation—A Frenchman's Discovery in Orthopædics—The Coming Generation in Germany—Canadian War Contingent Association—Pluck and Grit—Pensioners' Wards—Which Once Were Empty—"Wrens" in Hospital—The Merthyr Vale Miners' Decision—The Treatment of Tuberculosis in Peterborough—Poor-Law Women Visitors—The Salaries of a Municipal Hospital Staff—Bitter Cry of the Honey-lover—Blue Spirits—This Week's Drug Market—To Our Readers. | |
| **Madness in Wallpapers** ... ... ... ... | 223 |
| The Effects of Colour on Individuals. | |
| **A Case of Strangulated Femoral Hernia** ... | 224 |
| **A Public Medical Service in Being** ... ... | 225 |
| Leicester's Useful Example. | |
| **Village Practice.—III.** ... ... ... ... | 225 |
| An Autobiographical Fragment. | |
| **Carnegie Reports on Physical Welfare** ... | 227 |
| (4) Mother and Child in Scotland. | |
| **"A Nurse's Charter of Liberty"** ... ... | 228 |
| Shylock's Argument Out of Court. | |
| **The Matrons' and Sisters' Department** ... | 229 |
| Nursing Progress and its Developments: A Year's Work at the College of Nursing. | |
| Round the Hospitals. | |
| The Matrons' Private Conference—The Nurses on the *Kenilworth Castle*—Attempt to Coerce Poor-Law Matrons—Miss Baillie's Sensation—A Day Off Every Week for Probationers—Real Education for all Women. | |
| **The College of Nursing (Limited by Guarantee)** | 233 |
| Third Annual General Meeting. | |
| **Matrons' and Sisters' Appointments** ... .. | 236 |
| **The Higher Education of Nurses** ... ... | 237 |
| Its Relation to Applicants for Training. | |
| **Some Annual Meetings** ... ... ... ... | 239 |
| **The Editor's Letter-Box** ... ... ... ... | 240 |
| Nervous and Mental Disorders. | |
| **Parliament and the Profession** ... ... ... | iv |
| **Hospital and Institutional Results** ... ... | iv |
| **The Institutional Worker** ... ... (Suppl.) | 1 |
| A Register of Nurses' Ward Work: Answer to April Question. | |

